# Genetic Mapping of the *TtGL-2A* Long-Grain Locus in Tetraploid Wheat

**DOI:** 10.3390/plants15132076

**Published:** 2026-07-03

**Authors:** Jingjing Zuo, Tingting Kang, Xin Bai, Min Wang, Dan Tan, Xin Li, Linyi Qiao, Guiyun Yan

**Affiliations:** College of Agronomy, Shanxi Agricultural University, Taiyuan 030031, Chinaqiaolinyi@sxau.edu.cn (L.Q.)

**Keywords:** tetraploid wheat, grain length, bulked segregant analysis, genetic mapping, transcriptome sequencing

## Abstract

Tetraploid wheat (*Triticum turgidum*), the progenitor of common wheat, provides rich genetic resources for wheat genetic improvement. TDI-1 is a long-grain cultivated emmer wheat (*T. turgidum* ssp. *dicoccum*) accession collected in our laboratory. It was crossed with TDU-1, a short-grain durum wheat (*T. turgidum* ssp. *durum*) accession. The F_1_ hybrids exhibited heterobeltiosis (i.e., performance superior to the better parent) for grain length, thousand-kernel weight, and other kernel traits. In the F_2_ population, grain length showed a strong positive correlation with thousand-kernel weight (*r* = 0.77), and the ratio of plants with the short-grain parental phenotype, heterobeltiosis phenotype, and long-grain parental phenotype was approximately 1:2:1 (*χ*^2^ ≈ 0.697, *p* > 0.7), suggesting that a major locus plays a primary role in controlling grain length in this population. Combined with phenotyping of F_2:3_ families, homozygous long-grain and short-grain bulks constructed from F_2_ individuals were genotyped using bulked segregant analysis based on 120K-SNP array. The results showed that polymorphic SNPs were mainly concentrated on the short arm of chromosome 2A, leading to the inference that this region harbors a locus regulating grain length, tentatively designated *TtGL-2A*. Twelve SSR markers were developed on the short arm of chromosome 2A, mapping *TtGL-2A* to an 8.1 cM genetic interval between markers *2AS-280t26* and *2AS-280t29*, corresponding to the physical interval 121.2–158.8 Mb, with a LOD score of 12.3. Using the diagnostic marker *2AS-95t3* to genotype 253 wheat accessions, the proportion of long-grain allelic variation *TtGL-2A*_TDI in landraces, cultivars, and introduced lines is 40.27%, 75.86%, and 82.35%, respectively. Transcriptome sequencing of grains at 15 days post-anthesis from the parental lines TDI-1 and TDU-1 identified 11 differentially expressed genes within the *TtGL-2A* interval. These results lay a foundation for map-based cloning of *TtGL-2A* and provide an efficient marker for molecular breeding of wheat yield.

## 1. Introduction

Wheat (*Triticum aestivum*, *AABBDD*) is a major staple crop worldwide. Shiferaw et al. [1] predicted that global wheat production would need to increase by 60% by 2050 to meet the food demands of the growing population. However, the current rate of increase in wheat yields is gradually slowing down, and yield improvements based on conventional hybrid breeding techniques have reached a bottleneck [2]. A thorough analysis of the factors contributing to wheat yield, coupled with the extensive identification and utilization of yield-regulating loci from varieties, lines, landraces and even ancestral wheat species, has become a key objective of molecular breeding in wheat [3].

Grain phenotypes such as grain length (GL), grain width (GW) and grain diameter (GD) are closely related to thousand-grain weight (TGW) [4,5]. As one of the three major components of yield, TGW is largely determined by grain phenotypes such as grain length, grain width and grain diameter. Among these, wheat grain length shows a significant positive correlation with TGW. Grain length genes typically begin to be expressed 5–20 days after flowering in wheat [6,7]. Qin et al. [8] identified the major QTL for grain length, *qKl*-1BL, in a recombinant inbred population derived from the wheat varieties Kenong 9204 and Jing 411. This QTL exhibited LOD scores ranging from 2.58 to 11.76 and accounted for 4.76–21.15% of the phenotypic variation; yield assessment based on near-isogenic lines indicated that *qKl*-1BL increases thousand-grain weight by increasing grain length. Yang et al. [9] identified the thousand-grain weight QTL locus QTgw.cau-5A.2 in a recombinant inbred population derived from the local wheat variety Yanda 1817 and the high-yielding line Beinuo 6; through comparative genomic analysis, it was found that this locus corresponds to the region in rice where the *OsGL3* gene, which controls grain length, is located, thereby identifying the homologous gene *TaGL3-5A*; haplotype analysis indicated that the *TaGL3-5A-G* allele significantly increases grain length and thousand-grain weight in the Chinese wheat micro-core germplasm. Qu et al. [10] identified a major QTL, *QKL/TKW.sicau-SSY-2D*, simultaneously controlling wheat grain length and thousand-grain weight, in the mapping population of the wheat cross S849-8 × SY95-71, and the contribution of this QTL was further validated in Sichuan wheat germplasm. Niaz et al. [11] localized the major effect locus *Qgl1.hau.1B*, which promotes thousand-grain weight, in the genetic population of the wheat accession UC1110×PI610750. Furthermore, the *Qtgw.nau-4B* locus [12], the grain length locus *QGl.cau-2D.1* from synthetic hexaploid wheat [13], and the grain length locus *Qkl.sicau-BLE18-4A* from wheat mutants [14] have all been shown to significantly increase thousand-grain weight.

Tetraploid wheat (*T. turgidum*, *AABB*) is the ancestral species of common wheat and an important gene pool for wheat genetic improvement. There is considerable variation in grain length among different tetraploid wheat accessions [15]. The laboratory had previously collected a long-grain accession of cultivated dicoccum wheat (*T. turgidum* ssp. *dicoccum*), TDI-1, and crossed it with a short-grain accession of durum wheat (*T. turgidum* ssp. *durum*), TDU-1, to establish a genetic population [16]. In this study, we used the TDI-1 × TDU-1 F_2_ population and its F_2:3_ lines as experimental materials. Using bulk segregant analysis (BSA) and SNP array screening technology, we mapped the grain length locus; furthermore, by integrating transcriptomic sequencing data from parental grains, we analyzed the expressed genes within the grain length locus region, with the aim of laying the foundation for subsequent map-based cloning of the grain length gene and providing excellent germplasm resources and molecular markers for high-yield molecular breeding in wheat.

## 2. Results

### 2.1. Analysis of Parental and F_1_ Hybrid Representative Lines

**Figure 1:**
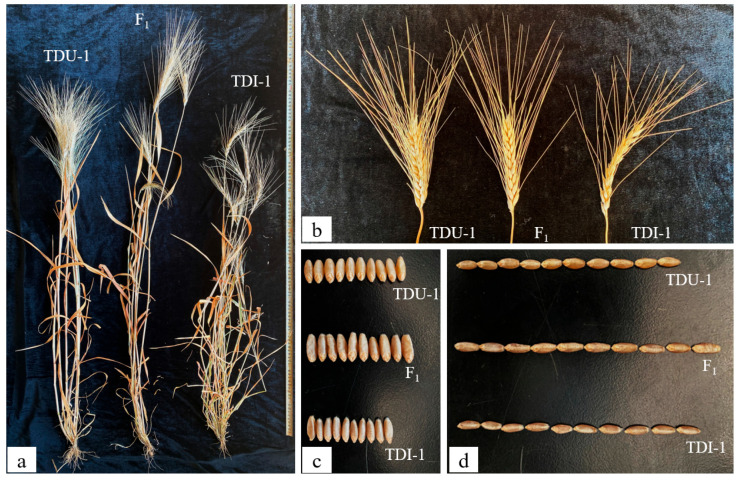
Phenotypes of tetraploid wheat parental lines and their F_1_ hybrids. TDI-1: long-grain cultivated emmer wheat; TDU-1: short-grain durum wheat; F_1_: hybrid progeny. (**a**) Plant phenotype; (**b**) spike phenotype; (**c**) grain width phenotype; (**d**) grain length phenotype.

Differences were observed in the agronomic and grain phenotypes of the tetraploid wheat lines TDI-1 and TDU-1 (Figure 1, Table 1). Compared with TDU-1, TDI-1 plants had a higher number of spikes (*p* < 0.01), longer grain length (*p* < 0.001) and higher thousand-grain weight (*p* < 0.001), but lower plant height (*p* < 0.001), fewer grains per spike (*p* < 0.01), narrower grain width (*p* < 0.01) and smaller grain diameter (*p* < 0.05); the difference in ear length between the two was not significant (*p* > 0.05). The number of ears and grains per spike in the F_1_ generation of the TDI-1 × TDU-1 hybrid fell between those of the parents, whilst ear length was similar to that of TDU-1; in contrast, the F_1_ generation plants exhibited both plant height and grain phenotypes superior to those of the parents, demonstrating heterobeltiosis (Figure 1, Table 1). Among the eight phenotypes measured, the coefficient of variation (*CV*) for grain length was the lowest in each parent (*CV* < 10%), and the difference between the two parents was the most significant (*p* = 0.00013); therefore, we focused on mapping the grain length phenotype.

### 2.2. F_2_ Population Genetic Model Analysis of Grain Length Phenotype

**Figure 2:**
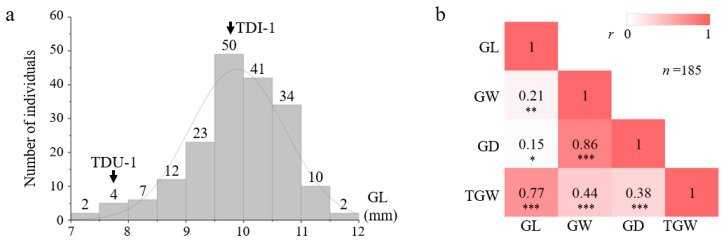
Grain phenotypes of the TDI-1 × TDU-1 F_2_ population. (**a**) The F_2_ grain length distribution with arrows indicating the parental means; (**b**) the correlation matrix of grain length (GL), grain width (GW), grain diameter (GD) and thousand-grain weight (TGW) with significance levels. *** for *p* < 0.001, ** for *p* < 0.01, and * for *p* < 0.05.

Grain traits were analyzed in the TDI-1 × TDU-1 F_2_ population. The results (Figure 2a) show that the grain length phenotype in the F_2_ population approximated a normal distribution, with grain length values for the short-grain parent TDU-1 lying to the left of the peak, and those for the long-grain parent TDI-1 corresponding to the peak. In the F_2_ population, there were 48 individuals with a grain length ≤ 7.53 mm to the left of the peak, 87 individuals with a grain length >9.87 mm to the right of the peak (heterobeltiosis phenotype), and 50 individuals with a grain length within the range 7.53–9.87 mm at the peak; the ratio of these three groups was approximately 1:2:1. A chi-squared test (*χ*^2^ ≈ 0.697, *p* > 0.7) confirmed that this pattern is consistent with the expectation of monogenic segregation. Therefore, a major locus, with a likely superdominant effect, primarily contributes to the grain length phenotype in this population. However, the heritability of grain length is not high in the F_2_ population (*H*^2^ = 0.54), with a skewness of −0.56 and a kurtosis of 0.32 (Table S1). Correlation analysis results show (Figure 2b) that grain length is strongly positively correlated with thousand-grain weight (*r* = 0.77) and weakly positively correlated with grain width (*r* = 0.21) and grain diameter (*r* = 0.15), whilst the correlation coefficients between grain width and grain diameter and thousand-grain weight are 0.44 and 0.43, respectively; it is inferred that the effect of grain length on increasing thousand-grain weight is stronger than that of grain width and grain diameter in the population.

### 2.3. BSA of Grain Length Phenotypes in the Population

**Figure 3:**
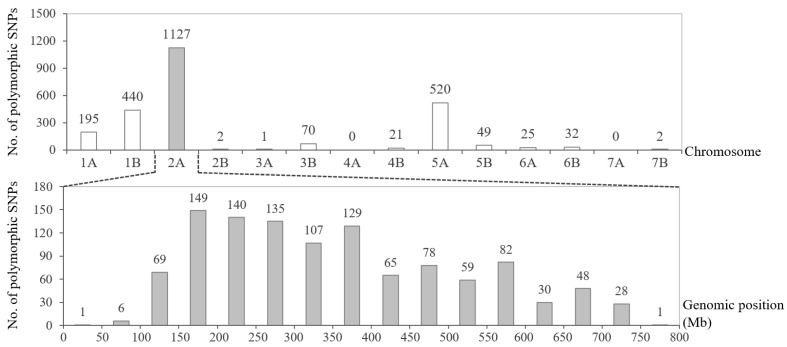
Distribution of polymorphic SNPs across the 14 chromosomes of tetraploid wheat by bulk segregant analysis.

Combining F_2:3_ family phenotypic data, individual DNA samples from homozygous long-grain and homozygous short-grain plants in the F_2_ population were selected to construct long-grain and short-grain pools. Genotyping of the mixed pools and parental lines was performed using an SNP array containing SNP markers evenly distributed across the 14 chromosomes of tetraploid wheat. The genotyping results showed that a total of 2484 markers exhibited consistent polymorphism between the long-grain parent TDI-1 and the short-grain parent TDU-1, as well as between the long-grain and short-grain pools. Among these, chromosome 2A harbored the largest number of polymorphic markers (1127), accounting for 45.37% of all polymorphic markers, and these were mainly concentrated within the physical region of 150–400 Mb on chromosome 2A, accounting for 58.56% of the polymorphic markers on chromosome 2A (Figure 3). Based on this, it is inferred that a grain length regulation locus exists on the short arm of chromosome 2A, provisionally named *TtGL-2A*.

### 2.4. Genetic Mapping of TtGL-2A

**Figure 4:**
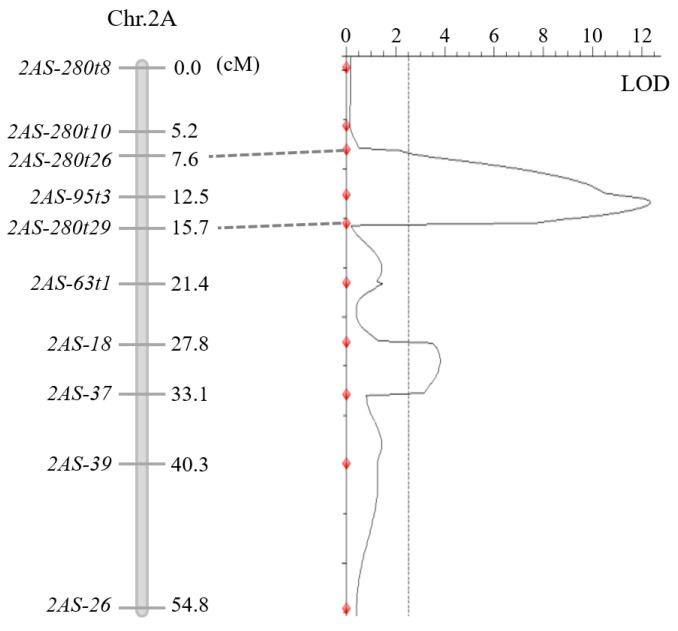
Genetic mapping of *TtGL-2A*. On the left are the molecular marker, and on the right are the corresponding genetic distance and LOD curve.

Twelve parental polymorphic SSR markers were developed on the short arm of chromosome 2A to amplify F_2_ population. In conjunction with the population’s grain length phenotype, 10 markers were ultimately integrated into the genetic map, and *TtGL-2A* was localized to a genetic region of approximately 8.1 cM between *2AS-280t26* and *2AS-280t29*, corresponding to the physical location 2A: 121.2–158.8 Mb. *TtGL-2A* has a LOD score of 12.3 and represents a major regulatory locus for grain length, with a phenotypic variation explained (*PVE*) value of 15.7%, and the additive effect is 0.49 mm. As *2AS-95t3* is located downstream of the LOD peak, it was selected as a diagnostic marker for *TtGL-2A* (Figure 4). Moreover, next to *TtGL-2A*, a locus with small effect (LOD = 3.71) was also detected between *2AS-18* and *2AS-37* (Figure 4).

### 2.5. Analysis of Allele Variation Distribution Frequencies

**Figure 5:**
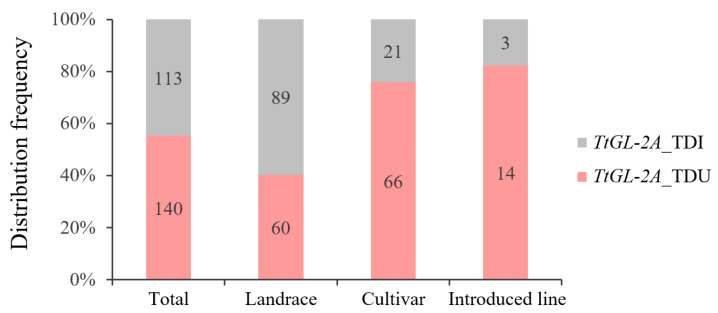
Distribution of *TtGL-2A* allele variation in wheat germplasm.

Using the *TtGL-2A* diagnostic marker *2AS-95t3* to amplify 253 wheat accessions, the results showed that 140 accessions (44.66%) carried the long-grain allele *TtGL-2A*_TDI, whilst 113 accessions (55.34%) carried the short-grain allele *TtGL-2A*_TDU. Among these, the proportion of the long-grain allele in landraces, cultivars, and introduced lines was 40.27%, 75.86% and 82.35%, respectively (Figure 5).

### 2.6. Differentially Expressed Genes Within the GL-2A Region

**Figure 6:**
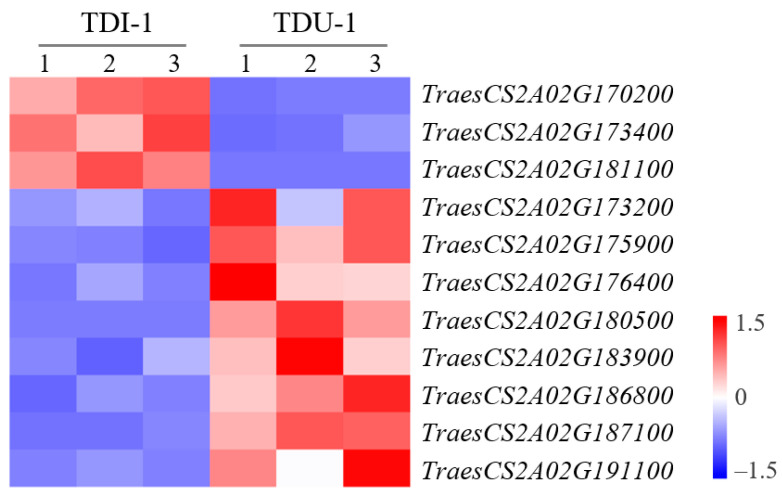
Differentially expressed genes within the *TtGL-2A* region based on the ‘Chinese Spring’ reference genome (IWGSC RefSeq v1.0). The annotation information of these genes is listed in Table 2.

As grain length genes begin to be expressed 5–20 days after flowering in wheat [6,7], this study performed transcriptomic sequencing on grain samples from the parental lines TDI-1 and TDU-1 15 days after flowering. Following assembly, alignment and differential analysis of the sequencing data, 234 high-confidence annotated genes were identified within the *GL-2A* region, of which 11 showed expression differences between the parental lines. Of these 11 DEGs, three were highly expressed in TDI-1, *TraesCS2A02G170200*, encoding adipocyte plasma membrane-associated protein (APMAP), *TraesCS2A02G173400*, encoding the gibberellin receptor GID1a, and *TraesCS2A02G181100*, encoding an F-box protein (FBP), whilst the remaining eight were highly expressed in TDU-1 (Figure 6, Table 2).

**Figure 7:**
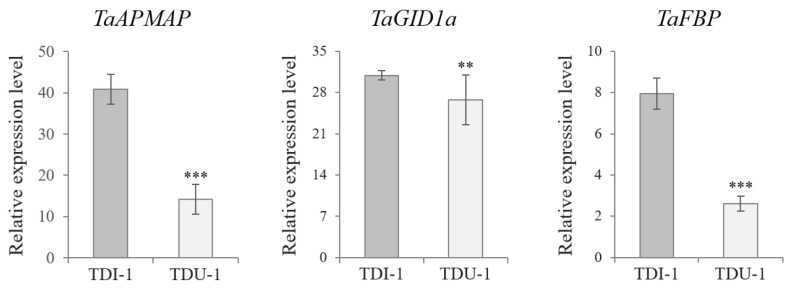
Relative expression level of three DEGs in growing grains at 15 days after flowering from TDI-1 and TDU-1. The bars indicate the standard error. ** indicates *p* < 0.01 and *** indicates *p* < 0.001, according to the *t*-test.

We validated the three DEGs that were highly expressed in TDI-1 by RT-qPCR, and the results showed that the relative expression levels of genes *TaAPMAP* (*p* < 0.001), *TaGID1a* (*p* < 0.01), and *TaFBP* (*p* < 0.001) were higher in the long-grained parent than in the short-grained parent, consistent with transcriptome sequencing data (Figure 7).

## 3. Discussion

### 3.1. Superdominance in Grain Length Phenotype of TDI-1 × TDU-1 Hybrid Progeny

Heterosis is a key theoretical foundation for maize and rice breeding, and its underlying mechanisms have been thoroughly elucidated [17,18]. As common wheat is a hexaploid, heterosis in hybrid progeny is typically attenuated, thereby limiting its application in breeding. In the mapping population for the wheat grain length locus QKL/TKW.sicau-SSY-2D, there are also instances where the phenotype of the offspring exceeds that of the parents [10], but as this population consists of recombinant inbred lines, the super parental phenomenon is not necessarily attributable to hybrid vigor. Compared with common wheat, hybrid vigor is more pronounced in tetraploid wheat [19]. In this study, significant super hybridism was observed in both the F_1_ generation plants and the F_2_ population derived from the cross between the tetraploid wheat lines TDI-1 and TDU-1. Furthermore, the grain length phenotypes of individual plants in the F_2_ population generally conformed to the segregation ratio expected for a single regulatory locus. This genetic pattern is typically associated with the complementary effects of favorable alleles in a heterozygous state, dose effects, or the synergistic action of regulatory elements. The heterobeltiosis observed for grain length in this study may stem from the long-grain allele carried by the parental line TDI-1, which, in a heterozygous state, is able to more efficiently activate pathways related to grain development, such as promoting cell division or elongation. Furthermore, the F_1_ generation also exhibited heterobeltiosis in terms of plant height, grain width and grain diameter, suggesting that the loci controlling grain length may be linked to other yield-related genes or exhibit pleiotropy. This study provides an ideal model for further elucidating the molecular mechanisms of hybrid vigor and offers a theoretical framework for high-yield breeding in hybrid wheat. While our results point to a single major locus, it is important to acknowledge that grain length is a typical quantitative trait influenced by multiple genes and environmental factors [4,5]. The 1:2:1 ratio observed here highlights the predominant effect of *TtGL-2A* in this specific genetic background, and the potential influence of minor QTLs cannot be excluded.

Notably, the heritability of grain length in the F_2_ population was not high, which suggests that heritability is not the only predictor for QTL detectability. The effect of a QTL (as reflected by its LOD score) primarily depends on the function of its causative gene(s), genomic background, and population structure, whereas heritability is influenced by the total genetic variance contributed by all genes as well as environmental variance. Many QTLs with high LOD scores but low heritability in populations have been successfully detected in multiple studies [20]. In our study, we hypothesize that the grain length locus may be predominantly governed by non-additive genetic effects, which is consistent with the observed heterobeltiosis phenotype in the F_2_ population.

### 3.2. The Breeding Value of the TtGL-2A Locus

Major QTLs controlling grain length have been reported on multiple wheat chromosomes, such as 1BL, 2D, 4B and 5A, whereas the effects of grain length loci on chromosome 2A are relatively minor. Examples include *QGl.ccsu-2A.1* and Q2A, which are located on the short arm, and *QGl.ccsu-2A.1*, *QGl.ccsu-2A.2* and *QL.cu.2A.1* [21,22,23]. Among these, *QGl.ccsu-2A.1* on the short arm originates from common wheat and is located within the *Xgwm497a–Xgwm95* interval (2A: 158.8–268.0 Mb), with a LOD score of 5.50 [21]; Q2A is derived from tetraploid wheat and is linked to *Xgwm473* (2A: 320.1 Mb), with a LOD score of 4.42 [22]. In this study, a major effect grain length locus, *TtGL-2A*, was identified within the physical region of 121.2–158.8 Mb on the short arm of chromosome 2A, with a LOD score of 12.3. As it differs from both *QGl.ccsu-2A.1* and Q2A, it is likely a novel locus.

The frequency of the favorable allele of *TtGL-2A* is significantly higher in Chinese wheat breeding lines (75.86%) and foreign introduced lines (82.35%) than in landraces (40.27%), suggesting that this locus may have undergone artificial selection during the breeding process. Furthermore, the frequency of this favorable allele is highest in introduced varieties, suggesting a possible association with the widespread cultivation of tetraploid wheat abroad (particularly in the Mediterranean region and North America). Furthermore, *TtGL-2A* shows a strong positive correlation with thousand-kernel weight (*r* = 0.77) and can be utilized for the genetic improvement of wheat yield. In this study, the *TtGL-2A* diagnostic marker *2AS-95t3* was developed for use in assisted selection during the breeding process.

In addition to the major peak on chromosome 2A, a secondary enrichment of polymorphic SNPs was observed on chromosome 5A, which may contain the known grain length locus *TaGL3-5A* [9]. Although its effect appears to be smaller than that of *TtGL-2A*, further analysis is needed to determine its contribution in this cross.

### 3.3. Candidate Gene Within the TtGL-2A Region

Eleven genes differentially expressed between the parental lines were identified within the *TtGL-2A* locus. Among these, the gene *TraesCS2A02G173400*, which encodes the gibberellin receptor GID1a, was upregulated in the long-grain parent TDI-1. It has been reported that GID1a influences the activation of downstream genes associated with cell elongation via the gibberellin signaling pathway, thereby regulating crop grain size [24,25]. In contrast, the F-box protein-encoding gene *TraesCS2A02G181100* and the F-box-like protein-encoding gene *TraesCS2A02G180500* exhibited opposite expression trends between the two parents. F-box proteins participate in the ubiquitin–proteasome pathway, regulating the cell cycle and hormone signal transduction, and their important role in controlling grain size has been confirmed in rice [26,27,28]. Furthermore, *TraesCS2A02G183900*, which encodes a GH3 protein, is upregulated in the short-grain parent TDU-1. GH3 proteins exert a negative feedback mechanism in the auxin signaling pathway; they catalyze the binding of indoleacetic acid to amino acids, thereby attenuating auxin signaling, and play a central role in IAA inactivation [29]. These DEGs can serve as candidate genes for subsequent map-based cloning and functional validation.

## 4. Materials and Methods

### 4.1. Experimental Materials

The long-grain cultivar TDI-1 of cultivated two-row wheat and the short-grain line TDU-1 of durum wheat were used for the construction of mapping populations and transcriptomic sequencing; the F_1_ hybrids of TDI-1 × TDU-1 (*n* = 9), the F_2_ population (*n* = 185 individuals) and its F_2:3_ families were used for phenotypic genetic analysis and molecular mapping. A total of 253 wheat accessions (comprising 149 landraces, 87 cultivars and 17 foreign introductions) were used to determine the frequency distribution of allelic variation at the grain length locus. All the aforementioned experimental materials are preserved by the Shanxi Provincial Key Laboratory of Germplasm Innovation and Genetic Improvement of Major Food Crops. The experiments were conducted in the greenhouse at the Dongyang Experimental Base of Shanxi Agricultural University from 2023 to 2025.

### 4.2. Phenotypic Assessment

In late October each year, hybrid progeny and their parental lines were sown in the greenhouse in plots 2.0 m long and 0.25 m wide, with 15 seeds planted at equal intervals in each row; for the F_2:3_ population, one row was planted per family, with 15 seeds sown. Conventional water and fertilizer management was applied. At wheat maturity (waxy stage), agronomic phenotypes were assessed, including plant height, number of spikes, spike length and number of spikelets per spike. After harvesting and threshing individual plants, the TPKZ3 intelligent seed inspection and analysis system (TopCloud Agriculture, Hangzhou, China) was used to measure grain phenotypic data such as grain length, width, diameter and thousand-grain weight. For each F_2:3_ family, 10 plants were randomly selected for measurement, and the mean values were calculated. Excel was used to analyze the phenotypic data, calculating standard deviation, coefficient of variation and significance of differences. Heterobeltiosis was determined using a one-sample *t*-test. Specifically, the F_1_ mean for each trait was compared against the better parent (e.g., TDI-1 for GL and TGW). A significant difference (*p* < 0.05) indicated heterobeltiosis. A chi-squared test was performed to test the goodness-of-fit to this ratio using the formula *χ*^2^ = *Σ* [(Observed − Expected)^2^/Expected], with degrees of freedom (*df*) = 2. The broad-sense heritability (*H*^2^) was estimated across environments by Genstat software (version 22), using the formula *H*^2^ = *V_G_*/*V_P_*, where *V_G_* and *V_P_* estimate genotypic and phenotypic variances, respectively.

### 4.3. BSA Based on Differential SNP Enrichment

Leaves were harvested from the seedling stage of the TDI-1 × TDU-1 F_2_ population and its parental lines, and genomic DNA was extracted using the CTAB method. Combining the grain phenotypic data from the F_2_ population and the F_2:3_ families, the BSA method was employed to select 20 homozygous long-grain individuals and 20 homozygous short-grain individuals from the F_2_ population. Their genomic DNA was mixed in equal proportions to establish a long-grain pool and a short-grain pool, respectively. The wheat 120K SNP array (TCUNI Technology Co., Ltd., Chengdu, China) was used to scan the parental lines and the pools, with two replicates of the experiment. Data inconsistent between replicates were filtered out, and SNP loci with consistent polymorphism between the parental lines and the pools were selected; the number and location of these loci on each chromosome were then counted. Chromosomal regions with the highest distribution of polymorphic SNPs were preliminarily identified as target regions. For the BSA using the 120K SNP array, the principle is that markers physically linked to the target gene will exhibit consistent polymorphism between the parental lines and the contrasting bulks. The region with the highest concentration of these consistently polymorphic markers is identified as the candidate region. This approach is well-suited for a major locus segregating in a simple 1:2:1 ratio, as it provides a robust and cost-effective alternative to high-depth sequencing for initial localization.

### 4.4. Genetic Mapping

Markers were developed and target loci mapped in accordance with the laboratory’s previously established method [30]. Referring to the genomic sequence of the wheat variety ‘China Spring’ (IWGSC RefSeq v1.0), Simple Sequence Repeat (SSR) loci were identified within the target region using SSRHunter 1.3 software, and genome-specific primers were designed (Table S2). Polymorphic SSR markers were screened from parental genomic DNA for use in amplifying the F_2_ population. Population genotyping data and grain length data were imported into Joinmap software (version 4.0); the Kosambi function was selected to construct a linkage map and perform genetic mapping of the grain length locus. The PCR reaction system was 10 μL: containing 5.0 μL PCR Mixture (Sangon Biotech Co., Ltd., Shanghai, China), 1.5 μL genomic DNA, 1.5 μL primers, and 2.0 μL ddH_2_O. Amplification products were electrophoresed on an 8% non-denaturing polyacrylamide gel, stained and visualized using the silver staining method, and the band patterns were recorded.

### 4.5. Transcriptome Sequencing

Grains were collected from the parental lines TDI-1 and TDU-1 15 days after flowering, with three biological replicates set for each time point. After constructing cDNA libraries from the grain samples, transcriptome sequencing was performed using the Illumina platform (BGI, Beijing, China). Data were processed according to the methods previously described by our laboratory [31]. Raw sequencing data were processed to remove low-quality or adapter-containing reads, yielding clean data for alignment against the Chinese Spring wheat reference genome (IWGSC RefSeq v1.0). Aligned reads were subsequently assembled and quantified. The FPKM value was used as the metric for gene expression levels. Differentially expressed genes (DEGs) were identified using the DESeq2 package. Genes with an absolute value of log_2_ (fold change) ≥ 1 and an adjusted *p*-value (FDR) < 0.05 were considered significantly differentially expressed. The FPKM (Fragments Per Kilobase of transcript per Million mapped reads) values were used as the metric for gene expression levels. The results were output in MeV format.

### 4.6. RT-qPCR

The sample backup used for transcriptome sequencing is also used for validation by RT-qPCR. Total RNA was extracted using an RNA extraction kit (Tianmo Bio, Beijing, China) and reverse-transcribed into cDNA using a reverse transcription kit (Takara Bio, Shiga, Japan). RT-qPCR was performed on the QuantStudio 3 Real-time PCR System (Applied Biosystems, Carlsbad, CA, USA), using the Premix Ex Taq II enzyme (Takara Bio, Shiga, Japan), and primer pairs for *TaAPMAP*, *TaGID1a*, *TaFBP*, and the internal reference gene *ACTIN* are listed in Table S2. Each reaction was repeated three times, and the results were analyzed using the 2^−∆*CT*^ and fold-change method, wherein the ∆*CT* was calculated using the following formula: ∆*CT* = *CT* (target gene) − *CT* (reference gene).

## 5. Conclusions

In this study, the F_1_ hybrids from the cross between tetraploid wheat TDI-1 and TDU-1 exhibited heterobeltiosis for grain length, thousand-kernel weight, and other kernel traits. In the F_2_ population, grain length showed a strong positive correlation with thousand-kernel weight (*r* = 0.77), and the ratio of plants with the short-grain parental phenotype, heterobeltiosis phenotype, and long-grain parental phenotype was approximately 1:2:1 (*χ*^2^ ≈ 0.697, *p* > 0.7), indicating that grain length is likely controlled by a single locus. Combined with phenotyping of F_2:3_ families, homozygous long-grain and short-grain bulks constructed from F_2_ individuals were genotyped using bulked segregant analysis (BSA) based on a 120K-SNP array. The results revealed that polymorphic SNPs were predominantly concentrated on the short arm of chromosome 2A, leading to the inference that a grain length regulatory locus resides in this region; this locus was tentatively designated *TtGL-2A*. Twelve SSR markers were developed on the short arm of chromosome 2A, and *TtGL-2A* was mapped to an 8.1 cM genetic interval between *2AS-280t26* and *2AS-280t29*, corresponding to the physical interval 121.2–158.8 Mb, with a LOD value of 12.3. Genotyping of 253 wheat accessions using the diagnostic marker *2AS-95t3* showed that the long-grain allele *TtGL-2A*_TDI accounted for 40.27%, 75.86%, and 82.35% in landraces, cultivars, and introduced lines, respectively. Transcriptome sequencing of grains at 15 days post-anthesis from the parents TDI-1 and TDU-1 identified 11 differentially expressed genes within the *TtGL-2A* interval. These results provide candidate genes for the map-based cloning and functional characterization of *TtGL-2A*. Collectively, the findings lay an important foundation for deciphering the molecular regulatory mechanisms of grain length and for high-yield molecular breeding in wheat.

## Figures and Tables

**Table 1:** Phenotypic values of parents TDI-1 and TDU-1 as well as their F_1_ generation.

Traits	Parents (*n* = 15)					F_1_ (*n* = 9)
	TDI-1	*CV* (%)	TDU-1	*CV* (%)	*p*	
Plant height (PH, cm)	98.32 ± 8.51	15	105.53 ± 7.58	11	0.00064	117.05 ± 6.68 *
Spike number (SN)	11.22 ± 5.51	34	7.57 ± 4.09	26	0.0025	8.35 ± 5.77
Spike length (SL, cm)	10.06 ± 1.88	18	8.97 ± 0.68	8	0.21	8.95 ± 1.26
Grain number per spike (GNPS)	31.43 ± 3.89	16	38.84 ± 2.56	11	0.0012	34.45 ± 4.37
Grain length (GL, mm)	9.87 ± 1.07	9	7.53 ± 0.16	3	0.00013	9.87 ± 1.23 *
Grain width (GW, mm)	2.46 ± 0.5	14	3.12 ± 0.22	7	0.0094	3.35 ± 0.40 *
Grain diameter (GD, mm)	4.46 ± 0.74	13	5.86 ± 0.23	5	0.017	5.97 ± 0.58 *
Thousand-grain weight (TGW, g)	52.26 ± 9.1	16	44.39 ± 10.85	19	0.00048	57.33 ± 9.64 *

Data are presented as mean ± SD; CV, coefficient of variation; * indicates heterobeltiosis.

**Table 2:** Annotation of differentially expressed genes within the *TtGL-2A* region.

Differentially Expressed Genes (DEG)	Genomic Location on chr.2A	Transcriptional Levels in TDI-1	Log2 (Fold Change)	Protein-Encoding Annotation
*TraesCS2A02G170200*	125,251,487–125,251,789	up	4.76	Adipocyte-like plasma membrane-associated protein
*TraesCS2A02G173400*	131,323,616–131,324,665	up	1.95	Gibberellin receptor GID1a
*TraesCS2A02G181100*	140,358,052–140,359,180	up	9.74	F-box protein
*TraesCS2A02G173200*	130,723,246–130,723,559	down	−1.90	Pectinesterase
*TraesCS2A02G175900*	134,880,371–134,880,658	down	−3.04	Methylesterase 11
*TraesCS2A02G176400*	136,051,206–136,051,218	down	−2.82	Glycerophosphodiester phosphodiesterase
*TraesCS2A02G180500*	139,509,183–139,510,317	down	−10.68	F-box-like protein
*TraesCS2A02G183900*	144,261,778–144,262,241	down	−1.26	GH3 protein
*TraesCS2A02G186800*	148,653,776–148,655,188	down	−1.86	HXXD-type acyltransferase
*TraesCS2A02G187100*	148,858,332–148,859,579	down	−3.81	Constitutive photomorphogenesis protein 10
*TraesCS2A02G191100*	158,040,432–158,040,743	down	−1.19	Acetylglutamate kinase

## Data Availability

The original contributions presented in this study are included in the article/Supplementary Materials. Further inquiries can be directed to the corresponding author.

## Supplementary Materials

The following supporting information can be downloaded at: https://www.mdpi.com/article/10.3390/plants15132076/s1, Table S1: Genetic parameters of grain length in TDI-1 × TDU-1 F_2_ population; Table S2: Primers used in this study.

## Abbreviations

The following abbreviations are used in this manuscript:

BSA	Bulked segregant analysis
CTAB	Cetyltrimethylammonium bromide
CV	Coefficient of variation
FPKM	Fragments Per Kilobase of transcript per Million mapped reads
GH3	Gretchen Hagen 3
LOD	Logarithm of odds
MeV	MultiExperiment Viewer
PCR	Polymerase chain reaction
QTL	Quantitative trait locus
SNP	Single nucleotide polymorphism
SSR	Simple sequence repeat
